# ThymicPeptides Reverse Immune Exhaustion in Patients with Reactivated Human Alphaherpesvirus1 Infections

**DOI:** 10.3390/ijms21072379

**Published:** 2020-03-30

**Authors:** Anna Hymos, Ewelina Grywalska, Janusz Klatka, Maria Klatka, Izabela Korona-Głowniak, Jacek Roliński

**Affiliations:** 1 Department of Otolaryngology and Laryngeal Oncology, Medical University of Lublin, 20-954 Lublin, Poland; 2 Department of Clinical Immunology and Immunotherapy, Medical University of Lublin, 20-093 Lublin, Poland; ewelina.grywalska@gmail.com (E.G.);; 3 Department of Immunology, St. John’s Cancer Centre, Lublin 20-090, Poland; 4 Department of Pediatric Endocrinology and Diabetology, Medical University, 20-093 Lublin, Poland; 5 Department of Pharmaceutical Microbiology, Medical University, 20-093 Lublin, Poland; izabelakoronaglowniak@umlub.pl

**Keywords:** herpes simplex, immune exhaustion, programmed cell death protein 1, thymic peptides

## Abstract

Recurrent infection with human alphaherpesvirus 1 (HHV-1) may be associated with immune exhaustion that impairs virus elimination. Thymic peptides enhance immune function and thus could overcome immune exhaustion. In this study, we investigated whether reactivation of herpes infections was associated with immune exhaustion. Moreover, we examined the impact of treatment with thymostimulin on the expression of programmed cell death protein 1 (PD-1) and its ligand (PD-L1) on T and B lymphocytes in patients suffering from recurrent HHV-1 reactivation. We also assessed the effector function of peripheral blood mononuclear cells (PBMCs) after stimulation with thymic peptides. We enrolled 50 women with reactivated HHV-1 infections and healthy volunteers. We measured the expression of various activation and exhaustion markers on the surface of PBMCs using flow cytometry. In ex vivo experiments, we measured the secretion of inflammatory cytokines by PBMCs cultured with thymostimulin. Compared with controls, patients with reactivated HHV-1 infections had increased percentages of CD3+ co-expressing CD25, an activation marker (*p* < 0.001). Moreover, these patients had increased percentages of CD4+ and CD8+ cells co-expressing the inhibitory markers PD-1 and PD-L1. In cultures of PBMCs from the patients, thymostimulin increased the secretion of interferon gamma (*p* < 0.001) and interleukin (IL)-2 (*p* = 0.023), but not IL-4 or IL-10.Two-month thymostimulin therapy resulted in no reactivation of HHV-1 infection during this period and the reduction of PD-1 and PD-L1 expression on the surface of T and B lymphocytes (*p* < 0.001). In conclusion, reactivation of herpes infection is associated with immune exhaustion, which could be reversed by treatment with thymic peptides.

## 1. Introduction

Human alphaherpesvirus 1 (HHV-1) is a common pathogen; nearly 80% of people aged 22–30 years have anti-HHV-1 antibodies, and the incidence increases with age [1,2]. Mucocutaneous lesions are the most common symptom of HHV-1 infections [3]. After the first infection, HHV-1 persists in neurons, and the infection can recur with or without symptoms [4,5]. Immunocompetent people combat HHV-1 infections without antiviral medications, whereas those with impaired immunity can have severe infections despite treatment [6].

Viral infections affect the immune function. T cells launch antiviral responses during acute infections, whereas chronic infection can cause T-cell exhaustion [7]. Exhausted T cells display a reduced production of antiviral cytokines, decreased cytotoxicity, and an impaired immediate response to previously encountered viral antigens [7].

Antigen stimulation activates lymphocytes, but it also increases the expression of programmed cell death protein 1 (PD-1), an inhibitory immune protein. The binding of PD-1 to programmed death ligand 1 (PD-L1) regulates the response to antigens [8]; it reduces the immune response and enhances tolerance to foreign antigens, increasing the risk of chronic infections [9].

Different thymic peptides enhance immune function [10]. Thymosin alpha and prothymosin alpha are among the best-studied thymic peptides [11]. Thymosin inhibits the replication of HHV-1 [12], and it increases the major histocompatibility complex(MHC)-mediated presentation of viral antigens on the cell surface for enhanced recognition of infected cells [13,14]. Moreover, thymosin alpha stimulates the production of interferon gamma (IFN-γ), interleukin (IL)-7, and IL-15, thereby increasing the number of T helper cells (Th), cytotoxic T cells, and natural killer (NK) cells [13,15].

The aim of this study was to measure the percentages of CD4+ and CD8+ cells co-expressing PD-1 and PD-L1 in patients with reactivated HHV-1 infections and to investigate the ability of immune cells to produce cytokines after stimulation with thymic peptides. We hypothesized that reactivation of herpes infections would be associated with the appearance of lymphocyte exhaustion markers on lymphocytes and that thymic peptides would stimulate antiviral responses in peripheral blood mononuclear cells (PBMCs).

## 2. Results

### 2.1. Participants

We enrolled 50 women with reactivated HHV-1 infections (age range: 19–66 years; mean age ± standard deviation (SD): 41.21 ± 12.42 years). Twenty volunteers served as a control group (age range: 18–69 years; mean age ± SD:42.87 ± 15.81 years). Table 1 presents the characteristics of the patients.

### 2.2. Frequencies of Basic Lymphocyte Subsets in Patients with Reactivated HHV-1 Infections and Controls

We assessed the frequencies of basic lymphocyte subsets in patients with reactivated HHV-1 infection and the control group. Patients exhibited significantly lower percentages ofCD19+ B cells than controls (*p* = 0.002, Table 2). Moreover, in the group of patients with reactivated HHV-1 infection, we found higher frequencies of CD4+CD25+highFOXP3+ T regulatory (Treg) cells and NK cells (*p* < 0.001, *p* < 0.025, respectively, Table 2).

### 2.3. Frequencies of CD25+, PD-1+, and PD-L1+ T and B Lymphocytes in Patients with Reactivated HHV-1 Infections and Controls

Patients with reactivated HHV-1 infections had a significantly higher percentage of CD3+CD25+ cells than did the controls (*p* < 0.001, Table 3). Moreover, the percentages of CD4+ cells and CD8+ cells co-expressing PD-1 were higher in patients with reactivated HHV-1 infections than in the control group (*p* < 0.001 for both comparisons). The percentages of CD4+ cells and CD8+ cells co-expressing PD-L1 were increased in patients with recurrent HHV-1 infections compared with the control group (*p* < 0.001 for both comparisons, Table 3).

### 2.4. Frequencies of PD-1+ and PD-L1+ B and T Lymphocytes in Patients with Reactivated HHV-1 Infections before and after 2 Months of Thymostimulin Therapy

Thymostimulin (TFX) is an approved medication for patients with impaired immune function. However, the impact of this substance on immune exhaustion, i.e., expression of PD-1/PD-L1 antigens, has yet to be established. In order to address this issue, we assessed the changes in the frequencies of PD-1+ and PD-L1+ T and B lymphocytes. All 50 patients were treated for 2 months with TFX in the following scheme: subcutaneous injection of one ampoule (10 mg) daily for 30 days, followed, over the next 30 days, by subcutaneous injection of one ampoule two times per week. During this period, all patients were free from HHV-1 infection reactivation, and we noticed a significant reduction in the percentages of CD19+PD-1+ B lymphocytes, CD4+PD-1+ T lymphocytes, CD8+PD-1+ T lymphocytes, CD4+PD-L1+ T lymphocytes, and CD8+PD-L1+ T lymphocytes (*p* < 0.001 for all comparisons, Table 4).

### 2.5. Stimulation of PBMCs with Thymic Peptides in Patients with Reactivated HHV-1 Infections

**Figure 1:**
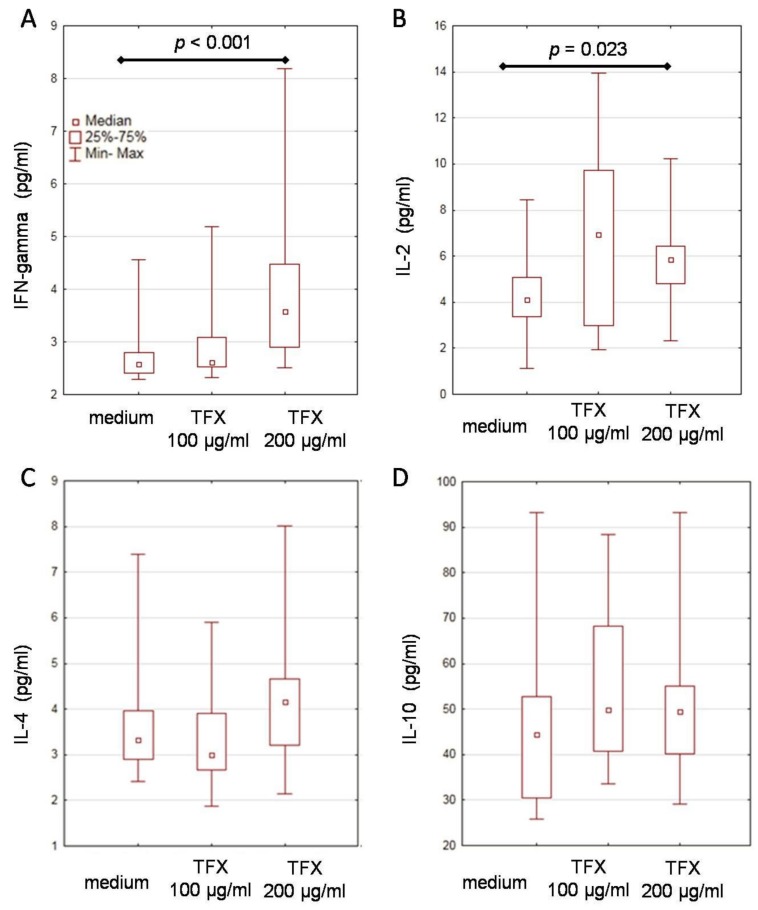
Secretion of IFN-γ (**A**), IL-2 (**B**), IL-4 (**C**), and IL-10 (**D**) by peripheral blood mononuclear cells from patients (*n* = 50) with reactivated HHV-1 infections. Boxes represent the interquartile range and error bars represent the range of observations. *p*-values are shown for significant differences. TFX: thymostimulin.

The concentrations of IFN-γ and IL-2 increased significantly in PBMC cultures supplemented with thymic peptides at the concentration of 200 µg/mL compared with concentrations in cultures without these peptides (*p* < 0.001 and *p* = 0.023, respectively; Table 3). The addition of thymic peptides to PBMC cultures did not significantly change the concentrations of IL-4 and IL-10 (Figure 1).

## 3. Discussion

Exposure of lymphocytes to viral antigens induces markers of activation on cell surfaces. Such activation markers are classified as early (CD69 and CD71) or late (CD25 and HLA-DR) markers depending on the time of stimulation [16]. T cells play an essential role in antiviral responses [17]. Jeon et al. showed that CD8+ T cells close to sensory neurons latently infected with HHV-1 expressed markers of immune activation, which indicated that lymphocytes come into contact with low levels of viral antigens during latency [18].Our study found that the percentage of CD3+ and CD4+ T cells co-expressing the CD25 activation antigen was higher in patients with reactivated herpes infections than in the control group. However, peripheral blood cells may not have reflected well the population of tissue-resident immune cells that come into contact with HHV-1 antigens.

Viral infections can strongly activate lymphocytes, which may lead to functional cell exhaustion [19]. Although virus-specific lymphocytes display activation markers, they are unable to kill infected cells or release antiviral cytokines [20]. The inability of cells to carry out effector functions may be one of the mechanisms underlying the silencing of antiviral responses, which impairs virus elimination [7,20,21]. The earliest sign of immune exhaustion is a decrease in immune cells’ capacity to secrete IFN-γ and IL-2 [8,22,23]. Yang et al. showed that the Us3 protein, which is expressed by HHV-1, inhibits T-cell activation and other immune responses [24].

Chronic antigen stimulation during viral reactivation may be associated with higher percentages of CD8+ T cells co-expressing PD-1. Khan et al. showed that exhaustion of CD8+ T cells can lead to recurrent symptomatic HHV-1 infections [21]. In addition, Mott et al. found higher levels of PD-1 transcripts during HHV-1 latency in neurons [25]. PD-1 expression inactivates CD8+ T cells in sensory neurons and impairs virus elimination [25]. Likewise, we found that the percentages of CD8+ and CD4+ cells co-expressing PD-1 were significantly higher in patients with reactivated HHV-1 infections than in the control group.

The interaction between lymphocytes and antigen-presenting cells (APCs) regulates immune responses. Co-stimulatory molecules determine whether T cells are activated or anergized [26,27]. PD-L1 transmits inhibitory signals to T cells that decrease their expansion, cytotoxicity, and cytokine production [28]. Blocking PD-L1 expression in APCs enhances lymphocyte proliferation and cytokine production by CD4+ T cells [29]. Importantly, an increased expression of PD-L1 in APCs was observed after HHV-1 infection [8]. In our study, the percentages of CD8+ and CD4+ cells co-expressing PD-L1 were significantly higher in patients with reactivated HHV-1 infections than in the control group. The simultaneously increased expression of PD-1 and PDL-1 suggests that there is a negative feedback control loop in the PD-1/PD-L1 axis during reactivation of herpes infections.

Sharpe et al. found an increased expression of PD-1 in CD4+ T cells in an animal model of keratitis [30]. In that model, blocking the PD-L1/PD-1 interaction improved both the proliferation and effector functions of HHV-1-specific CD4+ T cells [30]. Antoine et al. reported that CD4+ T cells specific to cytomegalovirus expressed activation markers during primary infection with the virus, but they had a decreased ability to produce IFN-γ, tumor necrosis factor alpha (TNF-α), and IL-2 [31].In our study, reactivation of herpes infection was associated with the presence of activated immune cells that, at the same time, expressed exhaustion markers. These findings indicate that the mechanisms that impair the immune response are similar for all herpes viruses, including HHV-1 and cytomegalovirus.

Immune exhaustion in chronic viral infections is most likely caused by alterations in many cell signaling pathways other than the upregulation of the PD-1/PD-L1 pathway. For example, infection with HIV or the hepatitis C virus increases the expression of a number of negative co-stimulatory molecules, including lymphocyte activation gene-3 (LAG-3), CD160, cytolytic T-lymphocyte antigen-4 (CTLA-4), T-cell immunoglobulin mucin-containing domain-3 (TIM-3), and tumor necrosis factor-related apoptosis-inducing ligand (TRAIL) [32,33]. Moreover, immune exhaustion might be related to mitochondrial dysfunction in immune effector cells [34]. Importantly, chronic viral infections might exhaust innate cells such as mucosal-cell-associated invariant T cells [35,36].

Matteucci et al. reported an enhanced antiviral response after the administration of thymosin alpha 1, which improves recognition of viral antigens and regulates cytokine gene expression [13,37,38]. In a study on patients with hepatitis B, Jiang et al. showed that treatment with thymosine alpha increased helper T cells’ production of IFN-γ, IL-2, IL-4, and TNF-α [39]. Decman et al. found that the addition of IFN-γ to cell cultures of neurons infected with HHV-1 blocked viral reactivation [40]. In our study, the thymic peptides contained in thymostimulin increased PBMCs’ production of IFN-γ and IL-2. This finding suggests that thymic peptides could increase the production of proinflammatory cytokines in patients with reactivated HHV-1 infections.

Our proof-of-concept study had limitations. The effect of thymostimulin was tested in vitro only, and the mechanism underlying thymostimulin-induced IFN-γ and IL-2 hypersecretion remains unknown. For example, it could involve foreign-antigen stimulation (thymostimulin is produced from bovine thymus extract). Experiments with recombinant human thymic peptides could help to test this possibility. Future studies should investigate the effect of thymostimulinon other diseases and healthy people. Moreover, further experiments should investigate whether reactivation of herpes infection is associated with impaired immune responses in general or with the antigens of HHV-1 only. Such experiments could determine whether thymostimulin improves antigen-specific immune responses or the immune response in general. Finally, we used CD25 as an activation marker of CD4+ cells; however, this approach is limited because a subset of these cells (~20%) are regulatory cells co-expressing FOXP3.

## 4. Materials and Methods

### 4.1. Patients with Reactivated HHV-1 Infections and the Control Group

Participants were patients who were referred to the Department of Clinical Immunology and Immunotherapy due to recurrent oral herpes. In practice, nearly all such patients are women, who seek medical help for oral herpes much more often than do men. We decided to include only women in order to have a more homogenous sample. All patients presented with reactivated HHV-1 infection at the time of the study. The control group included women scheduled for elective septoplasty due to nasal septum deviation or external nose deformity; we included women with no history of herpes virus infection. All controls were seronegative for HHV-1 and HHV-2. The exclusion criteria for both patients and controls were: treatment with an immunomodulating or hormonal medication, an infection within the 6 month period prior to enrolment, a confirmed allergy, an autoimmune disease, a history of blood transfusion, tuberculosis, cancer, and any condition impairing immunity. Peripheral blood samples were taken before treatment from 50 women with reactivated HHV-1 infections. Twenty volunteers served as a control group. The study was approved by the Medical Ethical Committee of the Medical University in Lublin (approval no., KE-0254/263/2014, 29 September 2014), and informed consent was obtained from each participant. The trial with TFX was open, single-arm, and uncontrolled. 

### 4.2. Material

Peripheral blood was collected in EDTA-coated tubes (10 mL) (Sarstedt, Nümbrecht, Germany) for immunophenotyping and isolation of PBMCs. Peripheral blood was also collected in tubes (5 mL) coated with a clotting activator to obtain serum for the measurement of anti-HHV-1 and HHV-2 antibodies.

### 4.3. Immunophenotyping

For immunophenotyping, flow cytometry was performed as described previously [41,42]. The cells were phenotypically characterized by incubation (20 min in the dark at room temperature) with a combination of fluorescein isothiocyanate (FITC), phycoerythrin (PE), and CyChrome-labeled monoclonal antibodies. Percentages of PD-1-positive and PD-L1-positive T and B lymphocytes were measured using a combination of the following monoclonal antibodies: CD45FITC/CD14 PE, CyChrome Mouse Anti-Human CD3, FITC Mouse Anti-Human CD19, FITC Mouse Anti-Human CD4, FITC Mouse Anti-Human CD8, PE Mouse Anti-Human CD279 (PD-1), and PE Mouse Anti-Human CD274 (PD-L1). Figure S1 shows CD4+ and CD8+ PD-1-positive (A) and CD4+ and CD8+ PD-L1-positive (B) T cells. The percentage of CD4+CD25+highFOXP3+ Tregs in the CD4+ T lymphocyte subpopulation was determined using the Human Treg Flow kit (FOXP3 Alexa Fluor 488/CD4 PE-Cy5/CD25 PE, BioLegend, San Diego, CA, USA). To identify the activated peripheral blood cells, we used the anti-CD25 PE-Cy5 mouse monoclonal antibody. Percentages of NK and natural killer T-like (NKT-like) cells were measured with flow cytometry using anti-CD3/FITC, CD16CD56/PE, and CD45/PerCPmonoclonal antibodies (BD Biosciences, San Jose, CA, USA), which allowed for the simultaneous assessment of CD3+T lymphocytes and NK (CD16+CD56+) cells. During analysis, the CD3+CD16+CD56+ (NKT-like cell) population was also determined. Monoclonal antibodies were purchased from BDBiosciences, USA. The “cleanness”of the lymphocyte gateway was evaluated by examining the distribution of cells in the coordinates of CD45CD14 (BDBiosciences, USA). The percentage of positive cells was measured froma cut-offset using an isotype-matched non-specific control antibody. Three-color immunofluorescence analyses were performed using a FACSCalibur flow cytometer (BD Biosciences, USA) equipped with a 488 nm argon laser. A minimum of 10,000 events were acquired and analyzed using CellQuest Software (BD Biosciences, USA). The percentages of cells expressing surface markers were measured. Background fluorescence was determined. The samples were gated on forwardscatter versus side scatter to exclude debris and cell aggregates.

### 4.4. Cytokine Secretion in Cultures of PBMCs Stimulated with Thymic Peptides

We measured the concentrations of cytokines secreted by cultured PBMCs from 15 patients with reactivated HHV-1 infections. PBMCs were obtained from peripheral blood by Ficoll-Hypaque density gradient centrifugation (2500 r/min for 20 min at room temperature). After collection, PBMCs were washed twice in phosphate-buffered saline. The viability of isolated cells was checked by trypan blue staining, and samples with a viability of<90% were excluded. PBMCs were stimulated with thymostimulin (Finepharm, Jelenia Góra, Poland), which is a bovine thymic extract containing proteins and polypeptides with a mass of 4000–12,000 Da.In Poland, thymostimulin is an approved medication for patients with an impaired immune function; however, the impact of this substance on immune exhaustion, i.e., expression of PD-1/PD-L1 antigens, has yet to be established.Cell cultures contained 10^6^ PBMCS/mL of medium. Two concentrations of thymostimulin were used: 100 µg/mL and 200 µg/mL. The cultures were performed in complete medium (RPMI-1640 supplemented with 10% fetal bovine serum (FBS), 200mM L-Gln(PAA Laboratories, Cölbe, Germany ), penicillin (100 IU/mL), streptomycin (50ug/mL), and neomycin (100ug/mL, Sigma Aldrich, Hamburg, Germany)). Cultures without thymic peptides were also performed. After 24 h at 37°C in 5% CO_2_, the cell cultures were collected. Cytokine concentrations in supernatants from cell cultures were then measured via enzyme-linked immunosorbent assays (ELISA).

### 4.5. Measurement of the Concentrations of IFN-γ, IL-2, IL-4, IL-10, anti-HHV-1 IgM Antibodies, anti-HHV-1 IgG Antibodies, anti-HHV-2 IgM Antibodies, and anti-HHV-2 IgG Antibodies

The concentrations of IFN-γ, IL-2, IL-4, and IL-10 were measured using commercially available ELISA kits (Human IFN-γ Quantikine (sensitivity, <8 pg/mL), Human IL-2 Quantikine (sensitivity, <7 pg/mL), Human IL-4 Quantikine (sensitivity, 0.03–0.22 pg/mL), and Human IL-10 Quantikine (sensitivity, 0.03–0.17 pg/mL), all purchased from RQD Systems, Minneapolis, MN, USA). The concentrations of anti-HSV1 IgM, anti-HSV1 IgG, anti-HHV-2IgM, and anti-HHV-2IgGantibodies were measured usingELISAs (IBL International, Hamburg, Germany). The cut-off value for anti-HHV-1IgM and anti-HHV-1IgGantibodies was >12 U/mL.The cut-off value for anti-HHV-2IgM and anti-HHV-2IgG antibodies was >11 U/mL. The concentrations of antibodies and cytokines were calculated based on standard curves. All ELISAs were performed according to the manufacturers’ instructions.

### 4.6. Statistical Analysis

The normality of the distribution of continuous variables was verified with the Shapiro–Wilk test. The groups were compared with the Mann–Whitney test or the Wilcoxon test. *p* < 0.05 was considered to be statistically significant. Statistica 12 software (StatSoft, Tulsa, OK, USA) was used for all analyses.

## 5. Conclusions

Reactivation of herpes infections is associated with an increased proportion of anergic immune cells, which impairs the immune response to the pathogen. The stimulation of PBMCs with thymic peptides increases the immune response, particularly the Th1-dependent response, which confirms the antiviral effects of thymic peptides. This approach may be useful to overcome anergy in patients with reactivated HHV-1 infections.

## Figures and Tables

**Table 1:** Characteristics of patients with reactivated human alphaherpesvirus 1 (HHV-1) infections and controls.

Variable	Statistics	Patients (*n* = 50)	Controls (*n* = 20)
Age (years)	median (min–max)	40.05 (19–66)	44 (18–69)
	mean ± SD	41.21 ± 12.42	42.87 ± 15.81
anti-HHV-1IgM, *n* (%)	Yes	8 (16%)	0
	No	42 (84%)	20 (100%)
anti-HHV-1 IgM, U/mL	median (min–max)	27.72 (12.11–62.7)	-
	mean ± SD	27.45 ± 18.33	-
anti-HHV-1IgG, *n* (%)	Yes	50 (100%)	0
	No	0	20 (100%)
anti-HHV-1 IgG, U/mL	median (min–max)	70.62 (22.41–1701.82)	-
	mean ± SD	167.09 ± 301.18	-
anti-HHV-2IgM, *n* (%)	Yes	0	0
	No	50 (100%)	20 (100%)
anti- HHV-2 IgG, *n* (%)	Yes	0	0
	No	50 (100%)	20 (100%)

**Table 2:** Subpopulation of lymphocytes in patients with reactivated HHV-1 infections before and after thymostimulin therapy (TFX),and the control group.

Variable		Patients with Reactivated HHV-1 Infections (*n* = 50)		Control Group (*n* = 20)	*p* (Patients before TFX vs. Controls)	*p* (Patients before vs. after TFX)
		before TFX	after TFX			
CD3+ T lymphocytes (%)	median (min–max)	69.62 (62.55–74.97)	73.66 (62.23-83.19)	71.94 (61.61–74.53)	NS	0.001
	mean ± SD	69.31 ± 3.35	73.31 ± 4.29	70.44 ± 3.53		
CD19+ B lymphocytes (%)	median (min–max)	10.44 (6.12–16.96)	15.31 (0.29–19.46)	13.62 (8.69–16.88)	0.002	<0.001
	mean ± SD	10.60 ± 3.22	13.95 ± 4.12	13.33 ± 2.46		
CD4+CD3+ T lymphocytes (%)	median (min–max)	39.16 (25.19–59.88)	40.25 (23.07–55.45)	38.34 (33.40–57.43)	NS	NS
	mean ± SD	39.92 ± 6.62	38.76 ± 9.36	41.06 ± 6.36		
CD8+CD3+ T lymphocytes (%)	median (min–max)	29.42 (11.17–49.29)	28.81 (9.01–52.22)	27.71 (12.78–37.16)	NS	NS
	mean ± SD	28.62 ± 7.07	31.04 ± 11.35	28.48 ± 6.10		
CD4+CD3+/CD8+CD3+ ratio	median (min–max)	1.29 (0.51–4.86)	1.49 (0.50–6.15)	1.34 (0.92–4.49)	NS	NS
	mean ± SD	1.58 ± 0.84	1.57 ± 1.04	1.58 ± 0.79		
NK cells (%)	median (min–max)	17.77 (10.16–27.72)	10.19 (3.43–18.56)	14.26 (9.16–24.08)	0.025	<0.001
	mean ± SD	17.51 ± 3.85	10.30 ± 3.96	15.09 ± 4.42		
NKT-like cells (%)	median (min–max)	4.19 (0.84–16.06)	4.31 (0.91–17.83)	3.29 (1.86–4.91)	0.048	NS
	mean ± SD	5.21 ± 3.35	5.43 ± 3.33	3.30 ± 1.02		
T regulatory cells (CD4+CD25+ FOXP3) (%)	median (min–max)	6.61 (3.13–16.85)	6.63 (3.11–13.66)	4.26 (3.23–7.45)	<0.001	0.020
	mean ± SD	8.32 ± 3.82	7.10 ± 2.72	4.45 ± 1.06		

SD: standard deviation; NS: not significant; TFX: thymostimulin therapy.

**Table 3:** Expression of the activation antigen CD25 and the immunosuppressive antigens PD-1 and PD-L1 on T and B lymphocytes in patients with reactivated HHV-1 infections before thymostimulin therapy (TFX) and the control group.

Variable		Patients with Reactivated HHV-1 Infections before TFX (*n* = 50)	Control Group (*n* = 20)	*p*
CD3+CD25+ T lymphocytes	median (min–max)	46.71(26.99–68.01)	37.06(18.65–47.13)	<0.001
	mean ± SD	47.17 ± 10.15	35.23 ± 8.33	
CD19+CD25+ B lymphocytes	median (min–max)	36.00(16.05–58.87)	18.64(10.88–27.50)	<0.001
	mean ± SD	36.59 ± 11.10	18.29 ± 4.16	
CD4+CD25+ T lymphocytes	median (min–max)	46.12(28.49–75.50)	35.79(30.64–47.50)	<0.001
	mean ± SD	49.01 ± 10.50	36.11 ± 3.97	
CD19+PD-1+ B lymphocytes	median (min–max)	6.35(1.91–25.47)	2.94(0.5–5.45)	<0.001
	mean ± SD	8.44 ± 5.99	3.05 ± 1.36	
CD4+PD-1+ T lymphocytes	median (min–max)	21.24(8.17–78.51)	8.73(6.19–11.34)	<0.001
	mean ± SD	25.03 ± 12.98	8.37 ± 1.46	
CD8+PD-1+ T lymphocytes	median (min–max)	17.73(8.40–40.73)	7.23(5.01–10.11)	<0.001
	mean ± SD	19.42 ± 7.34	7.32 ± 1.62	
CD19+PD-L1+B lymphocytes	median (min–max)	3.34(0.71–23.95)	2.39(0.12–4.70)	0.044
	mean ± SD	5.48 ± 5.23	2.33 ± 1.24	
CD4+PD-L1+ T lymphocytes	median (min–max)	14.67(6.78–63.81)	8.37(2.58–13.31)	<0.001
	mean ± SD	19.21 ± 12.78	7.99 ± 2.37	
CD8+PD-L1+ T lymphocytes	median (min–max)	11.43(2.35–33.67)	3.34(0.71–23.95)	<0.001
	mean ± SD	12.39 ± 6.80	3.36 ± 1.24	

SD: standard deviation; NS: not significant; TFX: thymostimulin therapy.

**Table 4:** Expression of the immunosuppressive antigens PD-1 and PD-L1 in T and B lymphocytes in patients with reactivated HHV-1 infections before and after 2 months of thymostimulin therapy (TFX).

Variable		Before TFX (*n* = 50)	After TFX (*n* = 50)	*p*
CD19+PD-1+ B lymphocytes	median (min–max)	6.35(1.91–25.47)	3.42 (0.83–14.36)	<0.001
	mean ± SD	8.44 ± 5.99	4.60 ± 3.37	
CD4+PD-1+ T lymphocytes	median (min–max)	21.24(8.17–78.51)	8.1 (1.14–27.96)	<0.001
	mean ± SD	25.03 ± 12.98	11.13 ± 7.9	
CD8+PD-1+ T lymphocytes	median (min–max)	17.73(8.40–40.73)	8.81(2.18–27.8)	<0.001
	mean ± SD	19.42 ± 7.34	8.96 ± 5.14	
CD19+PD-L1+ B lymphocytes	median (min–max)	3.34(0.71–23.95)	4.42(0.61–9.86)	NS
	mean ± SD	5.48 ± 5.23	4.57 ± 2.60	
CD4+PD-L1+ T lymphocytes	median (min–max)	14.67(6.78–63.81)	7.67(1.45–23.99)	<0.001
	mean ± SD	19.21 ± 12.78	8.23 ± 4.98	
CD8+PD-L1+ T lymphocytes	median (min–max)	11.43(2.35–33.67)	3.44(0.82–17.35)	<0.001
	mean ± SD	12.39 ± 6.80	4.97 ± 3.55	

SD: standard deviation; NS: not significant; TFX: thymostimulin therapy.

## Smentary Materials

Smentary Materials can be found at https://www.mdpi.com/1422-0067/21/7/2379/s1.

## Abbreviations

APC	antigen-presenting cells
CTLA-4	cytolytic T-lymphocyte antigen-4
ELISA	enzyme-linked immunosorbent assay
HHV-1	human alphaherpesvirus 1
IFN-γ	interferon gamma
IL	interleukin
LAG-3	lymphocyte activation gene-3
NK	natural killer cell
NKT-like	natural killer T-like cell
PBMC	peripheral blood mononuclear cells
PD-1	Programmed cell death protein 1
PD-L1	programmed death ligand 1
SD	standard deviation
TFX	thymostimulinum
Th	T helper cells
TIM-3	T-cell immunoglobulin mucin-containing domain-3
TNF-α	tumor necrosis factor alpha
TRAIL	tumor necrosis-factor-related apoptosis-inducing ligand
