# Supplementary figures and images for: ThymicPeptides Reverse Immune Exhaustion in Patients with Reactivated Human Alphaherpesvirus1 Infections
Source: Int J Mol Sci. 2020 Mar 30;21(7):2379. doi: 10.3390/ijms21072379 (PMC7178259; doi:10.3390/ijms21072379)

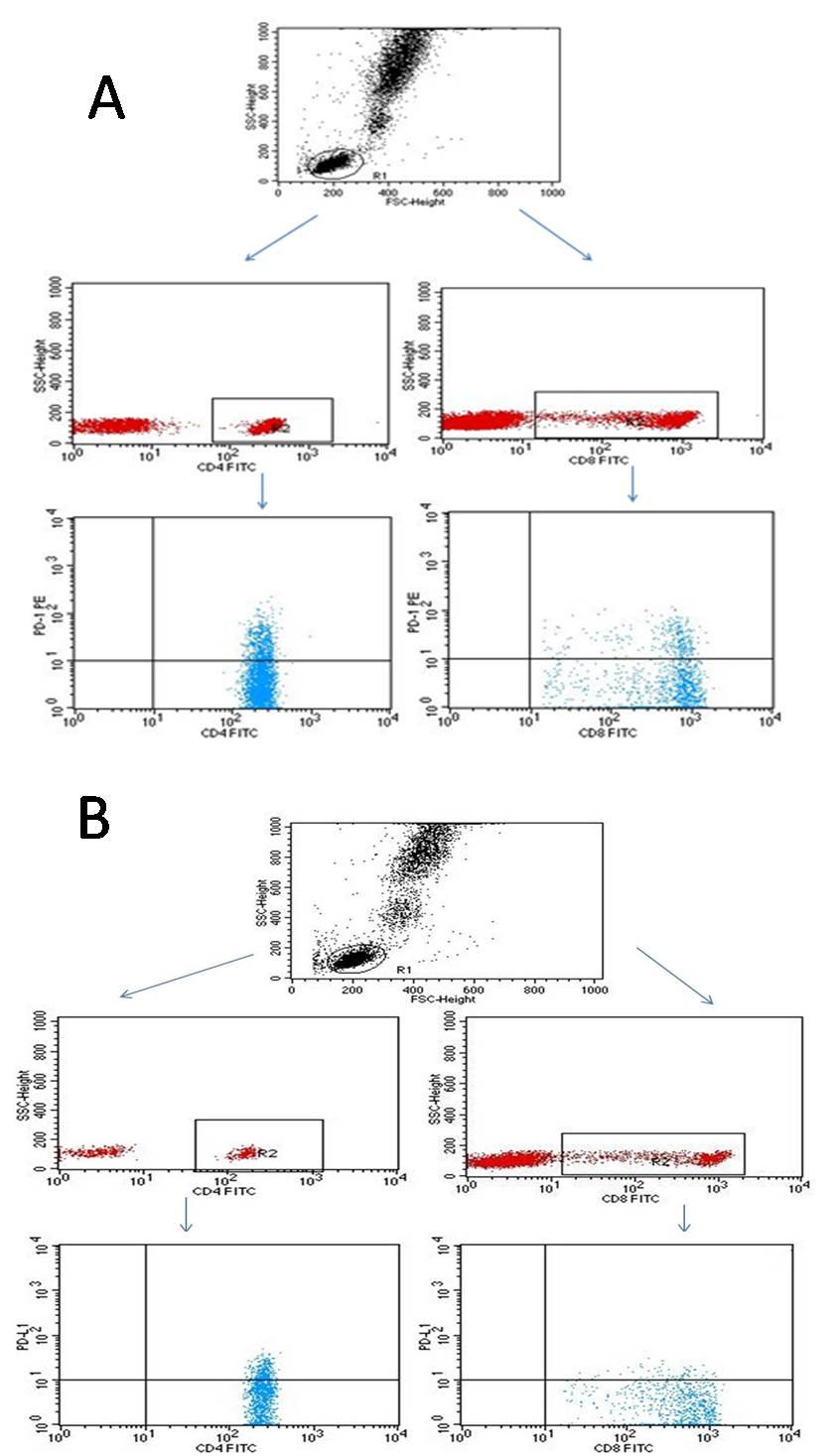

Supplement: Supplementary file 1 [file ijms-21-02379-s001.zip › ijms-740220-supplementary.jpg]
